# Iron-overloaded follicular fluid increases the risk of endometriosis-related infertility by triggering granulosa cell ferroptosis and oocyte dysmaturity

**DOI:** 10.1038/s41419-022-05037-8

**Published:** 2022-07-04

**Authors:** Zhexin Ni, Yangshuo Li, Di Song, Jie Ding, Shanshan Mei, Shuai Sun, Wen Cheng, Jin Yu, Ling Zhou, Yanping Kuang, Mingqing Li, Zailong Cai, Chaoqin Yu

**Affiliations:** 1 Department of Gynecology of Traditional Chinese Medicine, the First Affiliated Hospital of Naval Medical University, Shanghai, 200433 China; 2 Department of Assisted Reproduction, the First Affiliated Hospital of Naval Medical University, Shanghai, 200433 China; 3 Shanghai University of Traditional Chinese Medicine, Shanghai, 201203 China; 4 International Peace Maternity and Child Health Hospital, School of Medicine, Shanghai Jiao Tong University, Shanghai, 200025 China; 5 Department of Assisted Reproduction, Shanghai Ninth People’s Hospital, Shanghai Jiao Tong University School of Medicine, Shanghai, 200011 China; 6 Shanghai Key Laboratory of Female Reproductive Endocrine Related Diseases, Hospital of Obstetrics and Gynecology, Fudan University, Shanghai, 200000 China; 7 Department of Biochemistry and Molecular Biology, Naval Medical University, Shanghai, 200433 China

**Keywords:** Cell death, Infertility

## Abstract

Endometriosis (EMs) occurs in approximately 50% of women with infertility. The main causes of EMs-related infertility are follicle dysplasia and reduced oocyte quality. Iron overload occurs in ovarian follicular fluid (FF) of patients with EMs, and this condition is associated with oocyte maturation disorder. However, the underlying molecular mechanism remains largely unknown. In the present study, we identified the mechanism underlying ferroptosis in ovarian granulosa cells and oocyte maturation failure in EMs based on a retrospective review of in vitro fertilization/intracytoplasmic sperm injection-frozen embryo transfer outcomes in infertile patients with EMs. Mouse granulosa cells were treated with EMs-related infertile patients' follicular fluid (EMFF) in vitro. Western blot analysis, quantitative polymerase chain reaction, fluorescence staining, and transmission electron microscopy were used to assess granulosa cells ferroptosis. The effects of exosomes were examined by nanoparticle tracking analysis, RNA-seq, and Western blot analysis. Finally, the therapeutic values of vitamin E and iron chelator (deferoxamine mesylate) in vivo were evaluated in an EMs-related infertility model. Patients with ovarian EMs experienced poorer oocyte fertility than patients with non-ovarian EMs. We observed that EMFF with iron overload-induced granulosa cell ferroptosis in vitro and in vivo. Mechanically, nuclear receptor coactivator four-dependent ferritinophagy was involved in this process. Notably, granulosa cells undergoing ferroptosis further suppressed oocyte maturation by releasing exosomes from granulosa cells. In therapeutic studies, vitamin E and iron chelators effectively alleviated EMs-related infertility models. Our study indicates a novel mechanism through which EMFF with iron overload induces ferroptosis of granulosa cells and oocyte dysmaturity in EMs-related infertility, providing a potential therapeutic strategy for EMs-related infertility.

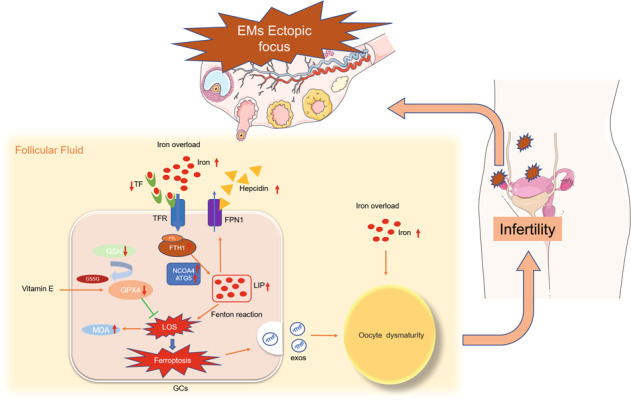

## Introduction

Infertility is a widespread global concern, and this condition is mainly caused by spousal factors, ovulatory dysfunction, and tubal disorders [1]. However, approximately half of infertile patients are diagnosed with endometriosis (EMs) [2]. Therefore, EMs-related infertility cannot be ignored. The mechanical disruption caused by pelvic adhesions and ovarian ectopic cysts in women with advanced EMs affects oocyte release, occludes fallopian tubes, and impairs the implantation environment of zygotes, which are major causes of female infertility [3, 4]. However, the mechanisms through which EMs leads to infertility in the absence of mechanical injury in patients with early or intermediate EMs have not been fully elucidated.

EMs is an estrogen-dependent disease that primarily affects pelvic tissues, including ovaries and fallopian tubes [5]. Iron overload caused by retrograde menstruation and periodic hemorrhage from ectopic lesions in the peritoneal fluid is an important factor in promoting the inflammatory microenvironment and adhesion formation in EMs [6, 7]. Iron overload in ovarian follicular fluid (FF) of patients with EMs impairs oocyte maturational development, but the exact mechanism has not been determined [8]. Oocytes affected by EMs show altered morphology, reduced cytoplasmic mitochondrial content, and reduced number of maturation [9]. Furthermore, granulosa cells in follicles are essential for the oocyte maturation process, and oocyte quality depends on interactions with surrounding granulosa cells [10]. Alterations of granulosa cells in EMs include decreased P450 aromatase expression and increased intracellular reactive oxygen species (ROS) levels [9]. However, the potential mechanisms underlying the interaction of iron-overloaded FF in EMs with granulosa cells and oocytes have not been determined.

Ferroptosis is a newly discovered non-apoptotic regulated cell death characterized by iron-dependent and lipid peroxidative accumulation [11]. Although ferroptosis has been implicated in the pathogenesis of some diseases, such as cancer, ischemic organ damage, neurodegeneration, and pulmonary fibrosis [12], limited mechanistic studies have focused on ferroptosis in EMs-related infertility. Ferroptosis showed a bidirectional role in the progression of EMs. Ectopic endometrial stromal cells (EESCs) suppress ferroptosis and promote the proliferation and migration by regulating the expression of related proteins and long non-coding RNA [13, 14]. Meanwhile, the occurrence of ferroptosis by local EESCs in contact with cyst fluid promotes angiogenesis in EMs [15]. However, the mechanisms of ferroptosis in granulosa cells and oocytes in EMs have not been fully determined.

In the present study, we aimed to explore the potential mechanism of granulosa cell ferroptosis in the FF of patients with EMs and its pathogenic effect on oocytes, and potential intervention strategies in vitro and in vivo.

## Results

### Ovarian endometriosis affects oocyte function

To investigate the effect of EMs on in vitro fertilization/intracytoplasmic sperm injection-frozen embryo transfer (IVF/ICSI-FET) outcomes and determine whether ovarian endometriosis (OE) is a major factor affecting IVF/ICSI-FET outcomes in EMs, we retrospectively analyzed 1712 infertile patients, including 385 in the OE group, 323 in the pelvic EMs (non-OE) group, and 1004 in the control group. Based on the baseline data sheet, higher estradiol (E_2_) levels and reduced number of antral follicles were observed in EMs patients compared with the controls; the number of antral follicles decreased in the OE group compared with the non-OE group (Table 1A). Furthermore, the number of punctured follicles, high-quality embryos, and cleavages were significantly smaller in the OE group than in the non-OE group (*P* < 0.05; *P* < 0.001; *P* = 0.05), except for poorer embryo transfer metrics in the EMs patients compared with the controls (Table 1B). Therefore, oocyte quality in the OE group was worse than that in the non-OE and control groups. However, no remarkable difference was observed in clinical pregnancy rates between the OE and non-OE groups (Table 1C), indicating that the effects of EMs on IVF/ICSI-FET outcomes are multifactorial and should be considered in addition to oocyte quality. Therefore, OE may contribute to the development of infertility directly by affecting oocyte function compared with non-OE.

**Table 1:** A Characteristics of baseline data, B oocyte retrieval cycle and embryo transfer index, and C neonatal outcomes.

	CON	EMs	P1	OE	Non-OE	P2
A						
Number of people	1004	708	/	385	323	/
Maternal age (years)	29.6 ± 3.5	33.4 ± 4.4	<0.001	32.7 ± 4.1	34.2 ± 4.6	<0.001
BMI (kg/m^2^)	21.0 ± 1.5	21.0 ± 3.0	ns	21.0 ± 3.0	21.1 ± 3.1	ns
Paternal age (years)	31.6 ± 4.6	35.0 ± 5.2	<0.001	34.2 ± 4.8	36.1 ± 5.4	<0.001
Infertility duration (years)	2.82 ± 2.2	3.32 ± 2.8	0.001	2.97 ± 2.4	3.73 ± 3.2	<0.001
Parity			<0.001			ns
0	997 (99.3)	664 (93.8)	/	357 (92.7)	307 (95.0)	/
>0	7 (0.7)	44 (6.2)	/	28 (7.3)	16 (5.0)	/
FET cycle rank	1.4 ± 0.8	1.8 ± 1.2	<0.001	1.7 ± 1.1	1.9 ± 1.3	0.01
FET endometrial preparation			<0.001			ns
Natural cycle	254 (25.3)	236 (33.3)	/	129 (33.5)	107 (33.1)	/
Artifcial cycle	750 (74.7)	472 (66.7)	/	256 (66.5)	216 (66.9)	/
Endometrial thickness (mm)	11.1 ± 2.3	10.8 ± 2.4	ns	10.9 ± 2.4	10.7 ± 2.3	ns
Endometrial thickness grouping			ns			ns
<8 mm	40 (4.0)	39 (5.5)	/	21 (5.5)	18 (5.6)	/
8~11 mm	516 (51.4)	390 (55.1)	/	204 (53.0)	186 (57.6)	/
>11 mm	448 (44.6)	279 (39.4)	/	160 (41.6)	119 (36.8)	/
Basal FSH (IU/L)	5.53 ± 2.0	7.49 ± 5.7	<0.001	7.46 ± 5.4	7.54 ± 6.0	ns
Basal LH (IU/L)	5.95 ± 6.2	6.17 ± 7.5	ns	6.47 ± 7.6	5.83 ± 7.3	ns
Basal E_2_ (pg/ml)	59.3 ± 61.0	74.6 ± 76.9	<0.001	79.6 ± 80.9	68.6 ± 71.5	ns
Number of antral follicles	10.3 ± 8.1	5.0 ± 5.8	<0.001	4.5 ± 5.4	5.5 ± 6.2	0.016

B						
Number of people	1004	708	/	385	323	/
Total number of follicles	5.5 ± 6.6	2.9 ± 4.2	<0.001	2.7 ± 3.6	3.2 ± 4.8	ns
Number of follicles >10 mm	1.5 ± 1.6	1.1 ± 1.2	<0.001	1.0 ± 1.0	1.1 ± 1.4	ns
Number of follicles >10 mm	1.1 ± 1.1	0.8 ± 0.8	<0.001	0.8 ± 0.8	0.9 ± 0.9	ns
Number of follicles punctured	18.9 ± 11.0	11.3 ± 9.2	<0.001	10.6 ± 9.1	12.1 ± 9.2	0.025
Number of retrieved oocytes	13.9 ± 7.9	7.9 ± 5.8	<0.001	7.6 ± 5.5	8.3 ± 6.1	ns
Rate of retrieved oocytes	0.75 ± 0.23	0.71 ± 0.23	0.004	0.71 ± 0.23	0.71 ± 0.23	ns
Total number of mature oocytes	11.5 ± 6.6	6.8 ± 4.6	<0.001	6.49 ± 4.1	7.08 ± 5.1	ns
Fertilization method			<0.001			ns
IVF	34 (3.4)	555 (78.4)	/	308 (80.0)	247 (76.5)	/
ICSI	922 (91.8)	108 (15.3)	/	59 (15.3)	49 (15.2)	/
IVE + ICSI	48 (4.8)	45 (6.4)	/	18 (4.7)	27 (8.4)	/
Total number of effective blastocysts	1.0 ± 1.4	0.6 ± 1.1	<0.001	0.58 ± 1.1	0.65 ± 1.1	ns
Total number of high-quality embryos	4.5 ± 3.2	3.3 ± 2.4	<0.001	2.97 ± 2.1	3.64 ± 2.7	<0.001
Total number of normal fertilized eggs	9.4 ± 5.6	5.9 ± 4.0	<0.001	5.67 ± 3.6	6.21 ± 4.4	ns
Total number of cleavages	9.2 ± 5.5	5.8 ± 3.8	<0.001	5.56 ± 3.5	6.13 ± 4.2	0.05
Number of embryos transferred			<0.001			ns
1	154 (15.3)	157 (22.2)	/	84 (21.8)	73 (22.6)	/
≥2	850 (84.7)	551 (77.8)	/	301 (78.2)	250 (77.4)	/
Embryo developmental stage at transfer			ns			ns
Cleavage stage	881 (87.7)	601 (84.9)	/	327 (84.9)	274 (84.8)	/
Blastocyst stage	123 (12.3)	107 (15.1)	/	58 (15.1)	49 (15.2)	/

C						
Number of people	1004	708	/	385	323	/
Number (rate) of clinical pregnancies	463 (46.1)	248 (35.0)	<0.001	145 (37.7)	103 (31.8)	ns
Delivery mode			ns			ns
Natural birth	141 (30.5)	60 (24.2)	/	36 (24.8)	24 (23.3)	/
Cesarean section	322 (69.5)	188 (75.8)	/	109 (75.2)	79 (76.7)	/
Gestational age (week)	38.1 ± 2.0	38.0 ± 1.9	ns	37.9 ± 1.9	38.3 ± 1.9	ns
Gestational age grouping			ns			ns
Very preterm birth (<32 weeks)	8 (1.7)	6 (2.4)	/	3 (2.1)	3 (2.9)	/
Preterm birth (<37 weeks)	55 (11.9)	33 (13.3)	/	24 (16.6)	9 (8.7)	/
≥37 weeks	400 (86.4)	209 (84.3)	/	118 (81.4)	91 (88.3)	/
Number of newborns			0.021			0.014
Singleton	339 (73.2)	201 (81.0)	/	110 (75.9)	91 (88.3)	/
Twin	124 (26.8)	47 (19.0)	/	35 (24.1)	12 (11.7)	/
Birth weight (g)	3139 ± 607	3140 ± 580	ns	3092 ± 627	3189 ± 576	ns
Birth weight grouping			ns			ns
Very low birth weight (<1500 g)	6 (1.3)	2 (0.8)	/	1 (0.7)	1 (1.0)	/
Low birth weight (<2500 g)	44 (9.5)	28 (11.3)	/	18 (11.7)	10 (9.7)	/
Birth weight (2500–4000 g))	398 (86.0)	208 (83.9)	/	121 (83.4)	87 (84.5)	/
High birth weight (>4000 g)	15 (3.2)	10 (4.0)	/	5 (3.4)	5 (4.9)	/
Birth defect	11 (2.4)	9 (3.6)	ns	7 (4.8)	2 (1.9)	ns
Neonatal diseases	13 (2.8)	6 (2.4)	ns	4 (2.8)	2 (1.9)	ns

P1, EMs group compared with CON group. P2, OE group compared with non-OE group. The data are expressed as mean ± SD, or *n* (%). ns indicates no significant difference between the groups (*P* > 0.05).

For twins, the weight of newborns born should first be counted. P1, EMs group compared with CON group. P2, OE group compared with non-OE group. The data are expressed as mean ± SD, or *n* (%). ns indicates no significant difference between the groups (*P* > 0.05).

### Iron overload in EMFF induces granulosa cell ferroptosis

Iron overload and deficiency of transferrin (TF) have been found in EMs-related infertile patients follicular fluid (EMFF) [8]. To further explore the effects of iron overload with EMFF on human ovarian granulosa cell function, we intervened KGN, a human ovarian granulosa cell line, with EMFF and follicular fluid in the control group (COFF) in vitro. Cell viability measurement experiments showed a significant reduction in the number of viable cells in the EMFF group compared with the COFF group at FF volume concentration of 20% (*P* < 0.001, *P* < 0.01, Fig. S1A, B). Subsequent experiments were performed on KGN cultured for 48 h with a medium containing FF at 20% volume concentration. Further investigation showed that the proliferation and migration activities of KGN were inhibited in the EMFF group (Fig. S1C, D). In addition, transcriptome analysis was performed on the two groups of KGN to evaluate differential gene expression in the EMFF group. A total of 682 mRNAs with significant differences in expression between the two groups were selected (differential fold > 1.5), including 344 upregulated and 338 downregulated mRNAs (Fig. S1E–H).

**Fig. 1:**
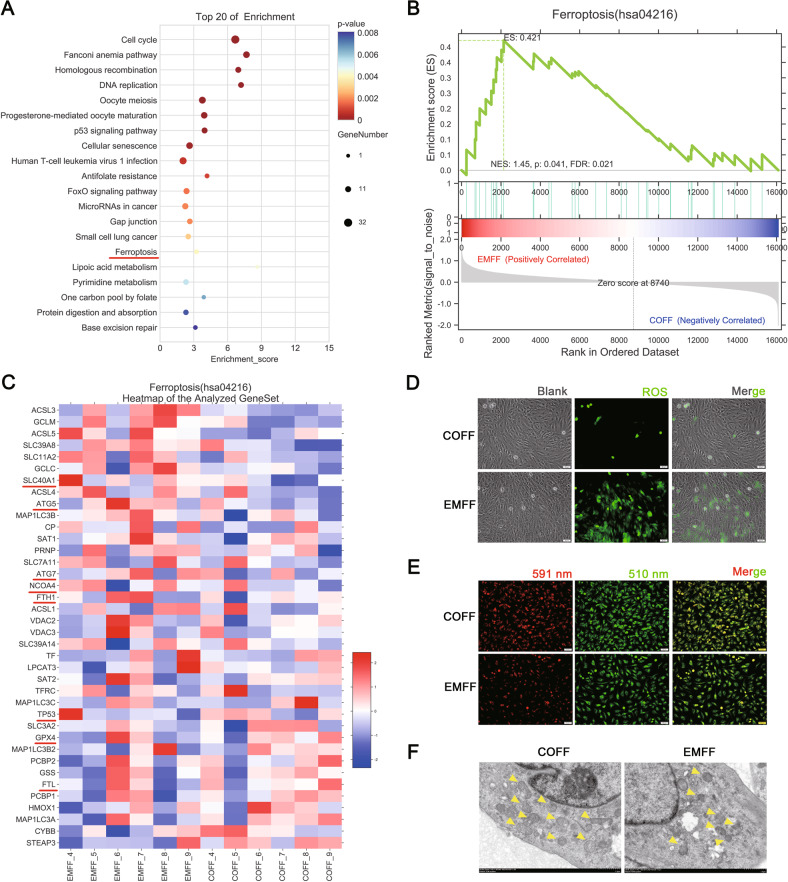
EMFF-induced granulosa cell ferroptosis. **A** KEGG enrichment top 20 bubble plot of KGN after EMFF treatment. The horizontal axis in the plot is the enrichment score. The larger the bubble, the greater the number of differential genes included. The lower the enrichment *P* value is, the higher the degree of significance. **B** GSEA analysis plot. Each line in the middle part of the figure represents one gene in the gene set and its ranked position in the gene list. The bottom part is a matrix of gene-phenotype associations, with red being positively correlated with EMFF and blue being positively correlated with COFF. FDR < 0.25 was set as credible enrichment. **C** Heatmap of transcript differential protein-coding genes (fold change > 2) between EMFF-KGN (*n* = 6) and COFF-KGN (*n* = 6). Red indicates relatively high expression protein-coding genes, and blue indicates relatively low expression protein-coding genes. **D** Representative images of ROS levels of EMFF-KGN and COFF-KGN under fluorescence staining. The green markers represent ROS level. Scale bar = 50 µm. **E** Representative images of C11-BODIPY (581/591) staining in EMFF-KGN and COFF-KGN groups. 591 nm represents the reduction state and 510 nm represents the oxidation state. Scale bar = 50 µm. **F** The mitochondrial morphology of COFF-KGN and EMFF-KGN was observed by TEM. Yellow arrows indicate mitochondrion. Scale bar = 1.0 µm.

KEGG enrichment analysis of the differentially expressed mRNAs revealed two pathways, including the ferroptosis and p53 signaling pathway, in addition to the related oocyte maturation pathway (Fig. 1A). To understand the potential effect of the ferroptosis pathway in the EMFF group, Gene Set Enrichment Analysis (GSEA) was performed to assess the expression levels of genes involved in the progression of ferroptosis in the EMFF group (Fig. 1B). Further analysis showed aberrant expression of iron autophagy-related genes, such as elevated expression of ATG5, nuclear receptor coactivator 4 (NCOA4), and ferritin heavy chain 1 (FTH1), and decreased expression of glutathione peroxidase 4 (GPX4) and tumor suppressor protein p53 (TP53, Fig. 1C). After subjecting both groups to treatment by immunofluorescence staining, the levels of ROS and lipid peroxidation increased in EMFF-treated KGN lines compared with those in the COFF group (Fig. 1D, E), and smaller mitochondria, higher membrane density, fewer mitochondrial ridges, and fragmented outer mitochondrial membrane were observed in the EMFF group compared with the COFF group under a transmission electron microscope (TEM, Fig. 1F). Therefore, KGN presents a risk of ferroptosis that impairs cellular functions after iron-overloaded EMFF treatment.

### Ferritinophagy is involved in iron-overloaded EMFF-induced ferroptosis in granulosa cells

**Fig. 2:**
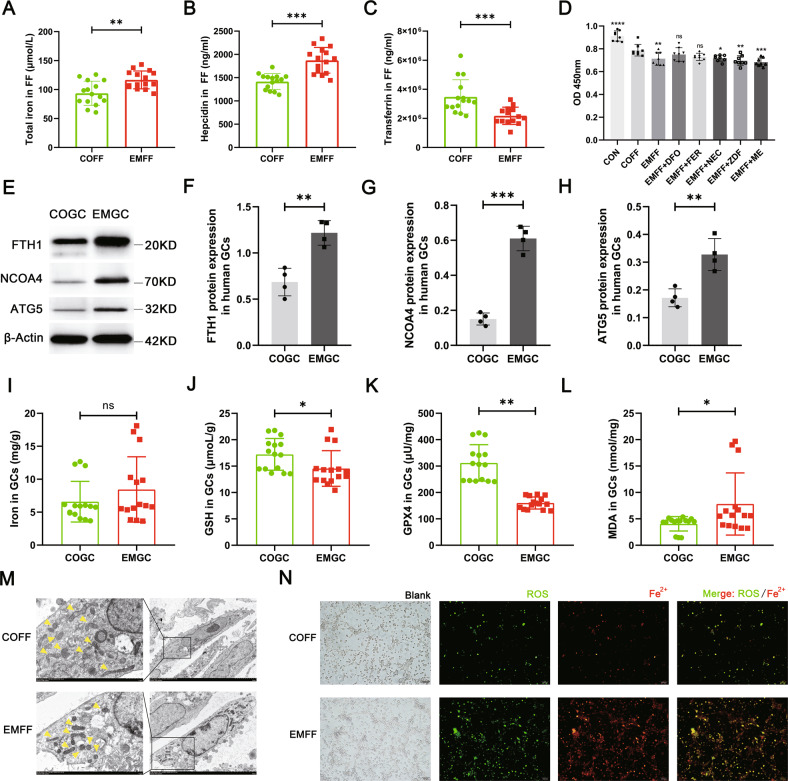
Iron-overloaded EMFF induced ferritinophagy-dependent ferroptosis in granulosa cells. **A**–**C** Levels of total iron, hepcidin, and transferrin in EMFF (*n* = 15) and COFF (*n* = 15). Data are expressed as means ± SD and analyzed by Student’s *t* test. **D** Results of mouse granulosa cells proliferation under different intervention conditions (each group in the figure is compared with COFF group). DFO, iron chelators; FER, ferroptosis inhibitor; NEC, necrosis inhibitor; ZDF, apoptosis inhibitor; ME, autophagy inhibitor. Data are expressed as means ± SD and analyzed by one-way ANOVA. **E**–**H** Comparison of ferritinophagy-related proteins FTH1, NCOA4, and ATG5 between human granulosa cells of infertile patients with EMs (EMGC) and of control group (COGC). The expression of β-actin was used as an internal control. Data are expressed as means ± SD and analyzed by Student’s *t* test. **I**–**L** Detection of ferroptosis-related indicators iron, GSH, GPX4, and MDA in COGC and EMGC. Data are expressed as means ± SD and analyzed by Student’s *t* test. **M** Representative images of the mitochondrial morphology of mouse granulosa cells intervened by COFF and EMFF were observed under TEM. Yellow arrows indicate mitochondrion. Scale bar = 1.0 µm. Scale bar = 5.0 µm. **N** Representative images of ROS and ferrous ion fluorescence staining after COFF and EMFF intervention in mouse granulosa cells. Scale bar = 100 µm. *P < 0.05, **P < 0.01, ***P < 0.001, ****P < 0.0001, and ns, no significance.

To further explore the mechanism of ferroptosis in granulosa cells induced by iron-overloaded EMFF, we studied clinical samples and examined the indicators of iron metabolism and ferroptosis in EMFF and granulosa cells from patients with EMs-related infertility. The levels of total iron and hepcidin increased, whereas TF expression decreased in the EMFF group compared with the COFF group (Fig. 2A–C). In addition, the expression of functional genes involved in ferroptosis and iron metabolism was abnormal in granulosa cells from patients with EMs (EMGC, Fig. S2). In particular, compared with granulosa cells in the control group (COGC), the expression of ferritinophagy-related genes that regulate ferroptosis was also abnormal in the EMGC group (Fig. S2). Notably, ferritin light chain (FTL) and FTH1, which constitute ferritin (FN), and NCOA4, ATG5, and ATG7, which mediate iron-selective autophagy (i.e., ferritinophagy), were all highly expressed in the EMFF group, whereas the expression of E3 ubiquitin ligase (HERC2) ubiquitinated NCOA4 by ubiquitin-proteasome system decreased (Fig. S2A–F). Furthermore, the expression levels of ferritinophagy proteins FTH1, NCOA4, and ATG5 in the EMGC group were significantly higher than those in the COGC group (Fig. 2E–H, *P* < 0.01, *P* < 0.001, *P* < 0.01). Although no significant difference was observed in the total iron levels between EMGC and COGC groups (*P* > 0.05), the contents of ferroptosis regulatory inhibitors, namely, glutathione and GPX4, decreased significantly, and the level of malondialdehyde (MDA), a metabolite of lipid peroxidation products, increased significantly in EMGC group (*P* < 0.05, *P* < 0.01, *P* < 0.05, Fig. 2I–L). Therefore, the ferritinophagy pathway may be a major factor that promotes the ferroptosis process of granulosa cells.

**Fig. 3:**
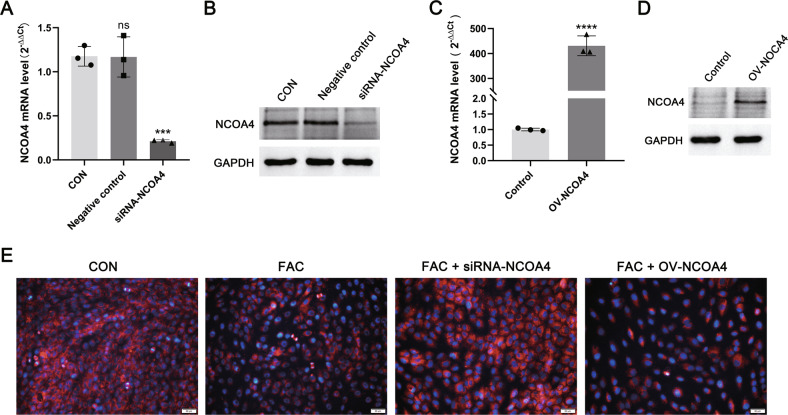
NCOA4-overexpressing and -silent. **A**, **B** Western blot analysis of KGN after siRNA silencing of NCOA4 (siRNA-NCOA4), control group, and negative control group. Expression of GAPDH protein was used as an internal control. **C**, **D** Western blot analysis of NCOA4-overexpression KGN (OV-NCOA4) and control groups. Expression of GAPDH protein was used as an internal control. **E** Representative images of lipid peroxidation and iron staining in KGN cells with silenced or overexpressed NCOA4. Blue represents iron, and red represents reduced state lipid peroxides. Scale bar = 50 µm. Data are expressed as means ± SD and analyzed by Student’s *t* test or one-way ANOVA. ***P < 0.001, ****P < 0.0001 and ns, no significance.

NCOA4 is a recently discovered autophagy cargo receptor that directly recognizes and binds FTH1, and then transports FN into autophagosomes for lysosomal degradation and iron release [16]. NCOA4 silencing inhibited ferritin degradation and ferroptosis, while the overexpression of NCOA4 increased ferritin degradation and promoted ferroptosis [17]. In the present study, we used ferric citrate (FAC) at concentrations close to human FF to intervene with KGN after NCOA4 silencing (siRNA-NCOA4) and overexpression (OV-NCOA4). Under immunofluorescence staining, ferric citrate increased the degree of cellular lipid peroxidation to diminish the red fluorescence belonging to the reduced state (Fig. 3). Silencing NCOA4 attenuated lipid peroxidation in KGN cells, whereas KGN after overexpression of NCOA4 further promoted lipid peroxidation (Fig. 3).

To explore whether the reduced granulosa cells bioactivity was caused by ferroptosis, we intervened mouse granulosa cells after EMFF treatment with different types of cell death inhibitors. The cell proliferative activity of granulosa cells in EMFF was reversed by the iron chelator (deferoxamine mesylate, DFO) and the ferroptosis inhibitor (Ferrostatin-1, FER), but not by the necrosis inhibitor (Necrostatin-1, NEC), the apoptosis inhibitor (Z-VAD-FMK, ZDF), and the autophagy inhibitor 3-methyladenine (ME, Fig. 2D). Furthermore, we verified in vitro that the intracellular ROS and ferrous ion (Fe^2+^) levels in mouse granulosa cells were higher in the EMFF group than in the COFF group (Fig. 2N), the mitochondrial volume was reduced, and the membrane color was deepened in the EMFF group (Fig. 2M). Therefore, NCOA4-mediated ferritinophagy is involved in EMFF-induced ferroptosis in granulosa cells.

### Granulosa cells in EMFF impair oocyte maturation via exosomes

**Fig. 4:**
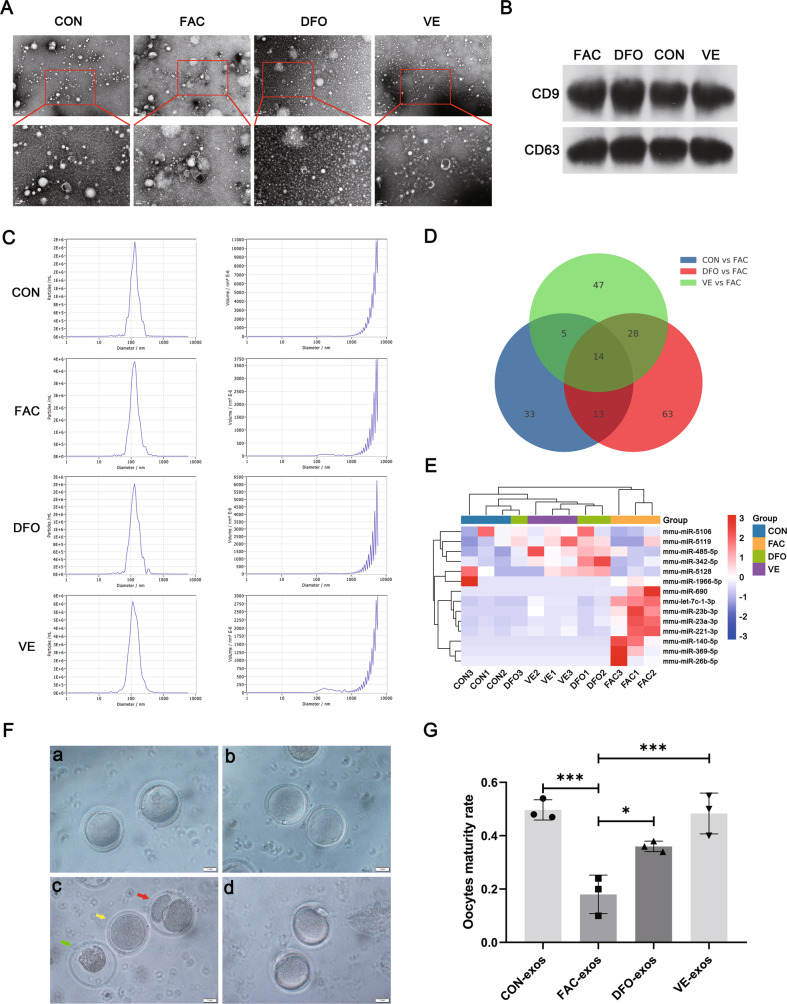
Characterization of exosomes from mouse granulosa cell and their effects on oocytes under different conditions. **A** Representative images of exosomes of mouse granulosa cells in control group (CON), iron overload group (FAC), iron chelators group (DFO), and vitamin E group (VE) were observed by TEM. Scale bar = 0.2 µm; Scale bar = 100 nm. **B** Western blot analysis of exosome-related markers CD63 and CD9 in different groups. **C** Nanoparticle tracking analysis of the size distribution and concentration of exosomes. **D** Venn diagram of differential miRNAs in three comparison groups. CON vs FAC includes differential gene expression of exosomal microRNAs between CON and FAC groups, DFO vs FAC includes differential gene expression of exosomal microRNAs between DFO and FAC groups and VE vs FAC includes differential gene expression of exosomal microRNAs between VE and FAC groups (*P* < 0.05, fold change ≥ 1.5). **E** Heatmap of the expression levels of the 14 differential miRNAs in each group from the (**D**) Venn diagram. Red represents relatively high expression and blue represents relatively low expression. **F** Representative images of morphological appearance of oocytes after intervention by exosomes under microscope. Both figures a and b were germinal vesicles stage oocytes. Red arrow indicates parthenogenetic activation, yellow arrow indicates germinal vesicle breakdown stage, and green arrow indicates dead oocytes in figure c. Figure d was the second meiotic metaphase awaiting fertilization stage oocytes. Scale bar = 1.0 mm. **G** Maturation rate of oocytes in each group after intervention of mouse granulosa cell exosomes treated using different methods. Data are expressed as means ± SD and analyzed by one-way ANOVA. *P < 0.05 and ***P < 0.001.

Exosomes, as small single-membrane secretory organelles derived from cells, have the same topology as cells and participate in intercellular communication [18]. Vitamin E (VITE) is a lipophilic antioxidant that can compensate for lipid peroxidation caused by GPX4 deficiency and further inhibit ferroptosis [19, 20]. The exosome samples all showed a clear vesicle profile under the negative staining field, and the particle size was consistent with the criteria of exosomes (Fig. 4A). The size of the particles in our samples was ~120 nm, which was consistent with the size of exosome diameter via nanoparticle tracking analysis (Fig. 4C). Exosome marker proteins, namely, CD63 and CD9, were obtained on the extracted samples from each group (Fig. 4B), and the extracted samples were determined to be exosomes.

To further explore the effects of exosomes derived from granulosa cells on oocytes, we intervened mouse oocytes with exosomes of mouse granulosa cells extracted from culture supernatants of different groups. Expulsion of the first polar body defines the hallmark of oocyte maturation, at which point the oocyte is arrested in the second meiotic metaphase awaiting fertilization (MII stage) [21]. The morphology of immature oocytes after the culture that appear mainly includes GV stage (oocytes with germinal vesicles), GVBD stage (oocytes with germinal vesicle breakdown), parthenogenetic activation (PA, formation of two equal-sized cells), and death [21]. Oocyte maturation rate was defined as the number of oocytes at the MII stage/the number of oocyte cultures (PA excluded). PA refers to the completion of development to the embryo without the action of a spermatozoon, stimulated by some physicochemical factors, in which the formed embryonic stem cell has the same totipotency and proliferative capacity as the embryonic stem cell formed by sperm-oocyte binding, making it capable of directed differentiation and development [22]. PA was therefore excluded when calculating maturation rates. To facilitate the understanding of the characteristics of different stages of oocytes, we illustrated the morphology of oocytes at different stages in Fig. 4F, where (a) and (b) are all GV stage oocytes; (c) red arrows are PA, yellow arrows are GVBD stage and green arrows are dead oocytes; (d) MII stage oocytes. The intervention of oocytes with exosomes in each group revealed a significant decrease in oocyte maturation rate in the iron overload group (FAC-exos, *P* < 0.001), which was reversed in the iron chelator (DFO-exos) and VITE groups (VE-exos, *P* < 0.05, *P* < 0.001); a more pronounced effect was observed after VE-exos intervention compared with DFO-exos (Fig. 4G and Table 2). Therefore, granulosa cells in iron overload undergo ferroptosis and release exosomes to impair oocyte maturation, and deferoxamine mesylate and VITE ameliorate exosomes of granulosa cell in iron overload to improve the maturation rate of mouse oocytes.

**Table 2:** Mouse oocyte maturation after exosome intervention.

Groups	Tests	GV	GVBD	PA	Death	MII	Total	Maturity rate
CON-exos	1	18	24	4	14	52	112	0.48
	2	15	35	7	18	61	136	0.47
	3	13	19	3	12	51	98	0.54
FAC-exos	1	14	41	6	31	10	102	0.10
	2	17	45	9	39	32	142	0.24
	3	11	39	7	29	20	106	0.20
DFO-exos	1	19	32	7	22	38	118	0.34
	2	15	35	8	28	44	130	0.36
	3	12	28	5	21	38	104	0.38
VE-exos	1	13	26	3	19	58	119	0.50
	2	14	33	8	23	46	124	0.40
	3	9	22	4	17	58	110	0.55

miRNAs are small non-coding RNAs that affect cell growth and metabolism by regulating gene expression and can be carried by exosomes to be consumed by neighboring or distant cells to regulate recipient cells [23]. To explore the differences of miRNAs from exosomes of granulosa cell in iron overload and under normal conditions, Fig. S3 shows the differential miRNA expression between the FAC and CON groups, a differential miRNA expression heatmap, and KEGG enrichment analysis of the differential miRNAs (differential fold > 1.5). A total of 65 differential miRNAs were discovered and predicted by target genes, which hit several signaling pathways, including calcium, MAPK, cell cycle, oocyte meiosis, and ferroptosis signaling pathway. Therefore, granulosa cells in iron overload may affect oocyte function by releasing exosomes containing abnormal miRNAs. We further performed intersection analysis of differential miRNAs (differential fold > 1.5) between the CON and FAC, DFO and, and VE and FAC groups and found 14 differential miRNAs contained by all three comparison groups (Fig. 4D). A clustered heatmap analysis of the expression of these 14 differential miRNAs among the four groups revealed that the iron overload group (FAC) was distinct from the three other groups (Fig. 4E). In comparison with the three other groups, the levels of five differential miRNAs, namely, mmu-miR-5106, mmu-miR-5119, mmu-miR-485-5p, mmu-miR-342-5p, and mmu-miR-5128 were lower, whereas the levels of nine differential miRNAs, namely, mmu-miR-1966-5p, mmu-miR-690, mmu-let-7c-1-3p, mmu-miR-23b-3p, mmu-miR-23a-3p, mmu-miR-221-3p, mmu-miR-140-5p, mmu-miR-369-5p, and mmu-miR-26b-5p were higher in the FAC group (Fig. 4E). Collectively, the in vitro results confirmed that iron overload caused the aberrant miRNA components in exosomes, which then interfered with mouse oocyte maturation by affecting the cell cycle, oocyte meiosis, and ferroptosis signaling pathway, while deferoxamine mesylate and VITE can effectively improve this situation.

### VITE improves ovarian function in mice with EMs combined with iron overload

**Fig. 5:**
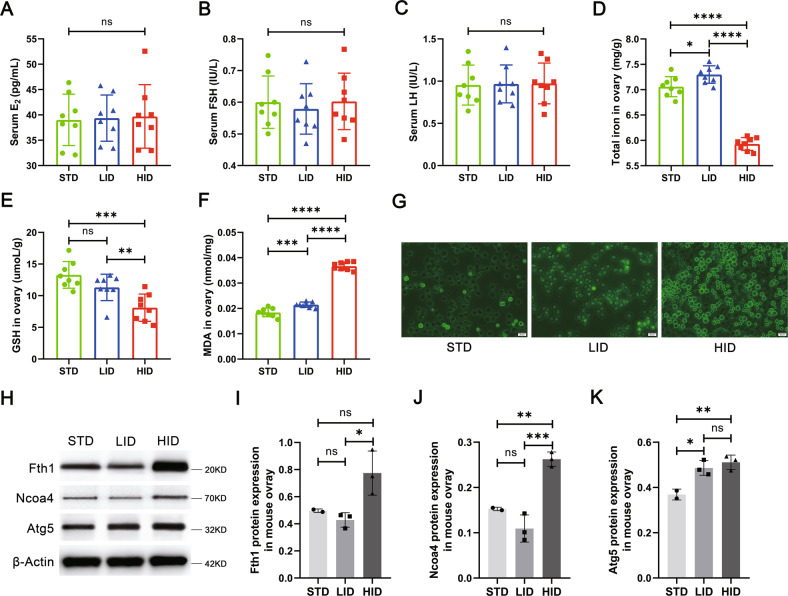
Construction of an iron overload mouse model. **A**–**C** Serum levels of E_2_, FSH, and LH in standard iron (STD), low iron (LID), and high iron (HID) diet feeding groups (*n* = 8). **D**–**F** Total iron, GSH, and MDA levels in the ovary tissues of mice in each group (*n* = 8). **G** Representative images of ROS fluorescence staining of ovarian mouse granulosa cells in three groups of mice. Scale bar = 20 µm. **H**–**K** Western blot analysis of ferritinophagy-related proteins, FTH1, NCOA4, and ATG5 in mouse ovary tissues in the STD, LID, and HID group. The expression of β-actin was used as an internal control. All data are expressed as means ± SD and analyzed by one-way ANOVA. *P < 0.05, **P < 0.01, ***P < 0.001, ****P < 0.0001, and ns, no significance.

To further validate the effect of iron overload on ovarian function in vivo, we fed mice with a standard iron diet (STD, *n* = 8), a low iron diet (LID, *n* = 8), and a high iron diet (HID, *n* = 8) group (Table S1). No significant abnormalities were observed in serum E_2_, follicle-stimulating hormone (FSH), and luteinizing hormone (LH) levels in the three groups (Fig. 5A–C), indicating that iron overload does not affect ovarian function through hormone level regulation. However, the levels of total iron, glutathione (GSH), and MDA were all abnormal in HID mouse ovarian tissues (Fig. 5D–F). Notably, the HID group had significantly lower iron levels and significantly higher MDA levels than the STD group (*P* < 0.0001, *P* < 0.0001), while the levels of iron and MDA increased in the LID group relative to the STD group (*P* < 0.05, *P* < 0.001). This phenomenon may have resulted from a self-protective mechanism initiated by the mouse ovary in the face of exogenous high iron intake but further induced an increase in MDA content. These results confirmed our successful construction of a mouse ovarian iron-overload model. Furthermore, ROS levels increased in granulosa cells from mice fed with HID (Fig. 5G), and the levels of the ferritinophagy-related proteins NCOA4 and ATG5 significantly increased in the HID group compared with the STD group (*P* < 0.01, *P* < 0.01, Fig. 5H–K), which was consistent with the expression of ferritinophagy-related proteins in EMGC.

**Fig. 6:**
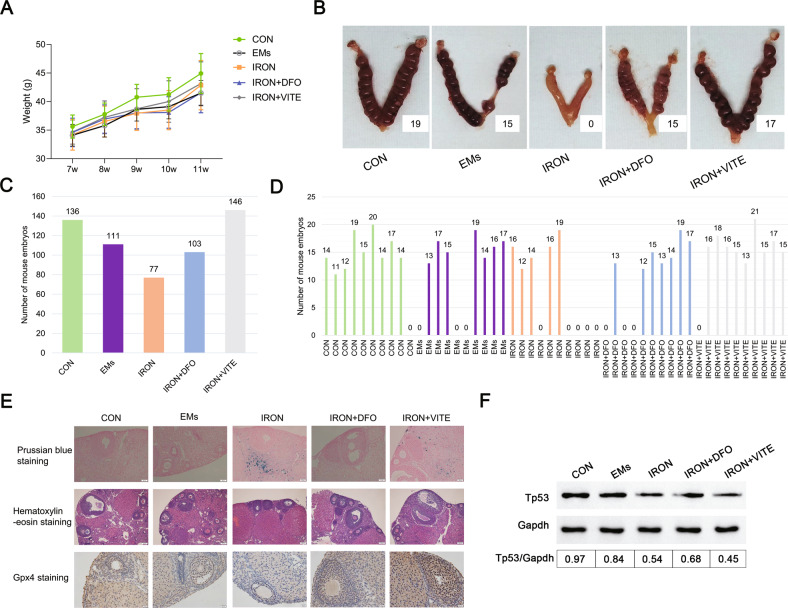
EMs combined with iron overload modeling affected mouse pregnancy. **A** The average body weight of the pregnant mice was calculated from control (CON, *n* = 10), EMs model (EMs, *n* = 10), EMs combined with iron overload model (IRON, *n* = 10), DFO treatment after IRON model (IRON + DFO, *n* = 10), and VITE treatment after IRON model (IRON + VITE, *n* = 10). **B** Representative photographs of the uterus after pregnancy in each of the five groups of female mice. **C**, **D** Total litter size and specific number of litters per mouse in five groups (*n* = 10). **E** For Prussian blue, H&E, and GPX4 immunohistochemical staining, ovarian tissues were observed from the five groups of mice. Scale bar = 50 µm; Scale bar = 200 µm; Scale bar = 20 µm. **F** Western blot analysis of TP53 expression in the ovarian tissues of mice in each group. Expression of GAPDH was used as an internal control.

To investigate the effects of EMs combined with iron overload on mouse litter size, we constructed five model groups, namely, control group (CON, *n* = 10), EMs group (EMs, *n* = 10), EMs with the high iron model group (IRON, *n* = 10), and deferoxamine mesylate and VITE intervention EMs with the high iron model group (IRON + DFO, *n* = 10 and IRON + VITE, *n* = 10, respectively). No differences in body weight were observed between the groups during pregnancy (Fig. 6A). We obtained 136, 111, 77, 103, and 146 litters from the five groups above (Fig. 6C) with average numbers of 13.6, 11.1, 7.7, 10.3, and 14.6, respectively. The IRON group had the lowest number of litters among the four groups. Furthermore, the frequency of mouse infertility was highest in the IRON group (50%), followed by the EMs and IRON + DFO groups (30%) and then the CON and IRON + VITE groups (10%, Fig. 6D). Representative pictures of each group are shown in Fig. 6B. Subsequently, we performed a histological examination of the ovaries of the female mice in each group. Prussian blue staining revealed that the iron-overloaded internal environment enabled the appearance of local iron accumulation in mouse ovarian tissue, while deferoxamine mesylate reduced local iron accumulation in the tissue, followed by the effect of VITE (Fig. 6E). H&E staining revealed that EMs and EMs with iron overload resulted in abnormal follicle development, while deferoxamine mesylate and VITE ameliorated the abnormal follicle development induced by high iron (Fig. 6E). Immunohistochemistry showed that the GPX4 level was reduced in the ovaries of IRON group, whereas both deferoxamine mesylate (DFO) and VITE restored GPX4 levels, and the effect of VITE was more obvious than that of deferoxamine mesylate (Fig. 6E). Considering that the mRNA expression of TP53 decreased in iron-overloaded KGN (Fig. 1C), we determined whether VITE and deferoxamine mesylate regulated ovarian function through the upregulation of TP53. However, only deferoxamine mesylate upregulated TP53 expression, whereas VITE caused a sustained decrease in TP53 expression (Fig. 6F), indicating that VITE improved GPX4 expression but not TP53, thereby protecting iron-overloaded mice from infertility.

## Discussion

EMs is a heterogeneous disorder with three phenotypes, namely, superficial peritoneal endometriosis, OE, and deep infiltrating endometriosis [24]. EMs adversely affects women’s reproductive capacity by acting on the pelvic cavity, ovaries, uterus, and fallopian tubes [3]. but the relationship between endometriosis phenotype and infertility has not been fully determined. The presence of OE per se is not associated with infertility, and surgical resection of OE deteriorates ovarian reservation [25, 26]. However, our retrospective analysis showed that oocyte quality was poorer in patients with OE than those with non-ovarian endometriosis (Table 1). Iron overload in the peritoneal fluid and FF of EMs patients is an important factor that leads to ectopic tissue proliferation and damage to oocytes [8, 27], but the specific mechanism in which iron overload in the FF affects granulosa cells and oocytes has not been fully elucidated. Considering that follicles close to the OE lesion tissues exhibit higher total iron levels and lower oocyte retrieval rates than follicles in healthy ovaries [28], iron overload in the OE may impair oocyte maturation and function by affecting granulosa cells close to the lesion and further mediate the release of abnormal exosomes of granulosa cells.

Ovarian granulosa cells are the largest cell population and major functional cells within the follicle, and follicle development is characterized by the rapid growth and proliferation of granulosa cells [29]. Oocyte activation and continued growth depend on the trophic and paracrine functions of their surrounding granulosa cells. However, granulosa cells in the ovaries of EMs patients appear abnormal and may be an important contributor to infertility. Excessive oxidative stress in granulosa cells of EMs-associated infertility causes cellular senescence and mitochondrial abnormalities [30], which is consistent with our findings. Iron overload in EMFF may be caused by the cyclic bleeding of local lesions and increased hepcidin levels in EMs-related infertile patients. Hepcidin is synthesized and secreted by the liver as a protein that plays an important role in body iron homeostasis. In the presence of iron overload in vivo, hepatic hepcidin secretion is increased, binds to cell surface iron exporter protein FPN1, promotes FPN1 endocytic degradation, and reduces intracellular iron outward transport [31]. Inflammation also increases hepcidin levels in the body [32], and EMs is a well-established inflammatory disease. Abnormalities in cellular senescence and ferroptosis signaling pathways occur after iron-overloaded EMFF intervention in the human granulosa cell line KGN, and elevated ferroptosis-related autophagy gene expression was observed by GSEA analysis. Considering that lipid peroxidation signaling is increasingly recognized as a core mediator of ferroptosis [33], elevated levels of iron, ROS, lipid peroxides, and MDA were detected, and shorter and condensed mitochondria were observed in KGN and granulosa cells after EMFF treatment, which is consistent with a previous study [11]. Furthermore, the key ferroptosis regulatory inhibitor GPX4 of the antioxidant system and its upstream regulatory target GSH expression were suppressed, and the cytotoxicity of EMFF against granulosa cells could be attenuated by an iron chelator and ferroptosis inhibitor but not by the apoptosis, necrosis, and autophagy inhibitor. Therefore, EMFF can induce granulosa cell death by ferroptosis.

Ferroptosis is an autophagy-dependent cell death [34]. In the ovary, an increase in ROS levels caused by hypoxia can induce autophagy in granulosa cells and oocytes. While autophagy removes damaged proteins and subcellular organelles to maintain cell survival, irreparable damage may induce cell death in the intrafollicular microenvironment [35]. The expression of iron selective autophagy-related genes and proteins was abnormal in granulosa cells from patients with EMs-related infertility (Fig. 2E–H). NCOA4 can selectively recognize the FTH1 subunit in FN and subsequently form the NCOA4-FN complex. The NCOA4-FN complex binds to ATG, thus forming the primary autophagosome, which subsequently develops into an autophagosome that degrades FN and releases free iron [34]. HERC2 can competitively bind NCOA4 with FTH1 and indirectly regulate ferritinophagy by ubiquitinating NCOA4 via the ubiquitin-proteasome system [36]. In the present study, the HERC2 gene expression level significantly decreased in EMGC (*P* < 0.05), whereas the FTH1 gene expression level was significantly increased in EMGC (*P* < 0.05). The role of NCOA4 in ferroptosis was determined by silencing the expression of NCOA4 by siRNA lentiviral transfection, and the result shows that an iron chelator-like effect was produced in the NCOA4-deficient KGN after ferric citrate (FAC) intervention (Fig. 3). Therefore, upon NCAO4 silencing, iron was stored in the form of FN, and ROS levels were consistent with the control group. Hence, the presence of iron overload in the FF of infertile patients with EMs can promote NCOA4 expression within granulosa cells, leading to active ferritinophagy, which further contributes to the development of ferroptosis by enhancing lipid peroxidation in granulosa cells.

The miRNAs contained in the exosomes released by granulosa cells after EMFF intervention were different from those in the control group, which may be the key factors of affecting oocyte quality. Exosomes are extracellular vesicles of endosomal origin with a size of 40–150 nm, and they play an important role in intercellular communication by delivering miRNAs, mRNAs, and proteins to recipient cells [37]. The miRNAs carried by exosomes are non-coding single-stranded RNA molecules with ~22 nucleotides in length encoded by endogenous genes, and they are involved in post-transcriptional gene expression regulation in animals and plants [23]. Exosomal miRNAs in granulosa cells under iron overload environment can regulate the gene expression of several signaling pathways. Dysregulation of calcium signaling pathways can induce ROS production, which is sufficient to cause oxidative stress in aging oocytes or trigger FAS ligand-mediated oocyte apoptosis [38]. After the MAPK signaling pathway is blocked, progesterone-induced MAPK activation is prevented, thereby inhibiting oocyte maturation [39]. Cell cycle signaling pathways and oocyte meiosis signaling pathways play an important role in oocyte maturation [40, 41]. Fourteen potential key miRNAs from exosomes can influence mouse oocyte maturation, including elevated maturation of miR-23a-3p/miR-23b-3p expression leads to perinatal oocyte loss [42], and upregulation of miR-221-3p expression involved in cell proliferation indicates decreased ovarian reserve [43]. These previous findings are consistent with the current findings. However, the downside is that these specific miRNAs have not been further analyzed to discover the downstream pathways they activate in oocytes.

Iron chelators and VITE can ameliorate infertility in mice with EMs combined with iron overload by affecting exosomes of granulosa cell secretion, illustrating the potential therapeutic role of iron chelators and VITE for infertile patients with EMs. Although both deferoxamine mesylate and the VITE inhibit ferroptosis, the molecular mechanisms by which they exert their effects differ among different studies. α-Tocopherol, a specific endogenous metabolite of VITE, can both specifically disrupt the propagation of the peroxidation chain and inhibit lipoxygenase expression [44]. Furthermore, ferroptosis induced by knockdown of GPX4 can be rescued by exogenous supplementation with VITE [45]. The iron chelator deferoxamine mesylate inhibits ferroptosis by reducing iron overload in cells [46]. We further verified that VITE and deferoxamine mesylate could improve GPX4 expression and decrease iron overload to improve the fertility of mice in EMs iron overload model mice (Fig. 6). Furthermore, deferoxamine mesylate increased TP53 expression in ovarian tissues of EMs iron overloaded mice, whereas VITE decreased TP53 expression. P53 has a dual role in the control of ferroptosis. TP53 can enhance ferroptosis by repressing the expression of solute carrier family 7 member 11 or by enhancing the expression of spermidine/spermine N1 acetyltransferase 1 and glutaminase 2 [12]. Meanwhile, p53 suppresses ferroptosis by directly inhibiting dipeptidyl peptidase 4 activity or inducing cyclin-dependent kinase inhibitor 1A/p21 expression [12]. The elevated TP53 expression is induced by deferoxamine mesylate, perhaps because the iron chelator mimics the hypoxic environment to activate the hypoxia regulatory capacity of p53 [47]. In conclusion, our work in this part validates the hazardous effects of an iron overload environment on oocyte development in animals and demonstrates the ameliorative effects of VITE in patients with EMs-related infertility. These findings will be of great reference for the clinical treatment of EMs-related infertility and also has implications for the treatment of other gynecological diseases under iron overload.

Recent studies have proposed ways to ameliorate EMs symptoms by promoting ferroptotic cell death in endometriotic lesions, but many practical issues should be considered. The environment of iron overload in the peritoneal fluid provides favorable conditions for the onset of ferroptosis, and the application of exogenous ferroptosis inducers promotes ferroptosis in EESCs [48]. However, safety issues for infertile patients with EMs should be further considered. Our study demonstrates the harm of an iron overload environment to the oocyte, and relevant studies have proposed that iron overload-induced ferroptosis impairs oocyte maturation and development [49]. Iron overload was thought to play an important contributory role in shaping the EMs inflammatory microenvironment and cell adhesion [6, 15, 27]. Combined with our findings, iron chelators, and VITE can be used for the treatment of EMs-related infertility. Ferroptosis inducers that target ectopic endometrial lesions can promote EESC ferroptosis for the treatment of EMs without harming oocytes.

In conclusion, we report a novel mechanism of oocyte damage in EMs-related infertile patients. The mechanism is that iron-overloaded FF induces granulosa cell ferroptosis and release exosomes of granulosa cell containing abnormal miRNAs to impair oocyte maturation. VITE and iron chelators can effectively ameliorate the symptoms.

## Subjects and methods

### Patients and sample collection

Baseline data sheet were collected from 1712 women, who underwent IVF/ICSI-FET between January 2010 and December 2018, in the assisted reproduction center of the hospital (Table 1). The inclusion and exclusion criteria for patients with EMs-related infertility and control subjects are shown in Supplementary material. After oocyte retrieval, FF was collected from EMs-related infertile patients and control females because of male infertility undergoing assisted reproduction. They were transferred to the laboratory to isolate FF and granulosa cells, obtaining EMFF, COFF, and granulosa cells.

### Mice models and mouse granulosa cell extraction

Female Kunming (KM) mice were obtained from Beijing Vital River Laboratory Animal Technology Co., Ltd (Beijing, China). After 1 week of adaptive feeding, experiments were carried out in strict accordance with the guidelines of the center for animal experimentation of Navy Medical University. The construction of animal models of iron overload ranging from high iron feed modeling to intraperitoneal administration of iron dextran has been reported and used in liver disease, cardiomyopathy, intracerebral hemorrhage, and hemochromatosis studies [50–53]. The experimental animal feed for the iron overload mouse model (Table S1) was synthesized by Jiangsu Medicience Biological Medicine Co., Ltd (Jiangsu, China). Mice were randomly assigned into STD (*n* = 8), LID (*n* = 8), and HID (*n* = 8). After mice were fed a specific diet for 8 weeks, normal female mice and a model of iron overload in female mice were constructed.

The modeling process of EMs mice has been reported [54]. Briefly, each mouse was injected subcutaneously with 2 μg β-estradiol benzoate solution (Sigma, #E2758) on days 1, 4, and 7. Endometrial fragment transplantation was performed on day 8. After setting up the blank control group (CON, *n* = 10), EMs mice were randomly divided into four groups (each group, *n* = 10). Except for the EMs group (*n* = 10), the three other groups were intraperitoneally injected with iron dextran (250 μg/g, Shanghai Yuanye Bio-Technology Co., Ltd, China, #9004-66-4) every 2 days for 3 consecutive weeks. On the second day after iron dextran intraperitoneal injection, two of the three groups were intraperitoneally injected with iron chelator deferoxamine mesylate (1 μg/g, MedChemExpress, USA, #HY-B0988) and VITE (50 μg/g, Solarbio, Beijing, China, #V8011) by gavage. Iron dextran treatment was stopped after three weeks, and deferoxamine mesylate and VITE intervention were extended for another week. The EMs group was treated with the same volume of normal saline by intraperitoneal injection and gavage. Twenty 12-week-old male Kunming mice were ordered in advance. Ten female mice from each group were selected for co-caged experiments. Daily citrulline feeding was initiated with males 1 week before co-caging, and males were pre-caged with females 1 day before co-caging to prevent mating. In the morning of the co-caging day, the female mice were administered with vaginal smears, and the female and male mice in the estrous period were co-caged with the ratio of one male mouse and two female mice in the evening. Sperm was found in vaginal smear the next morning considered to be successful for co-caged. Vaginal smears and co-caging were repeated until all female mouse vaginal smears see sperm. All female mice were sacrificed by ether anesthesia at no more than 18 days after the first female pregnancy. The mice's uterus was dissected, and the number of embryos in the uterus was counted.

Mice were intraperitoneally injected with pregnant horse serum gonadotropin (10 IU, Ningbo Sanseng Pharmaceutical Co., Ltd, Zhejiang, China). After 48 h, the mice were sacrificed under ether anesthesia, and then bilateral ovaries were removed. After stripping of ovarian adipose tissue, FF mixture was collected. EDTA free trypsin (0.25%, 1 ml, GIBCO, USA, #15050-065) was subsequently added, and then the mixture was placed in a 5% CO_2_ incubator at 37 °C for 30 min. Next, we filtered the FF mixture through a 70 μM cell strainer (BD Falcon, USA), and then centrifuged at 1000 rpm for 5 min. The supernatant was discarded, and the granulosa cells were obtained.

### Cell culture and viability measurements

KGN was obtained from Otwobio Biotech (Guangzhou) Inc. (China) and validated for authentication using the short tandem repeat (STR) method. KGN were maintained in Dulbecco’s modified Eagle’s medium (DMEM, HyClone, USA, #SH30022.01) medium (Invitrogen) containing 10% (*v*/*v*) fetal bovine serum and 1% (*v*/*v*) penicillin/streptomycin. Mouse granulosa cells and human granulosa cells were added with DMEM/F12 medium containing 10% (*v*/*v*) fetal bovine serum and 1% (*v*/*v*) penicillin/streptomycin, and volumes of medium added were adjusted for cell counts. After stimulation of indicated solution and inhibitors, the supernatant was removed and then rinsed thrice in phosphate-buffered saline (PBS, HyClone, #SH30256.01B). Approximately 110 μL of working solution containing 10 μL of cell counting Kit-8 (YOBIBIO, Shanghai, China, #U22-001A) and 100 μL of DMEM/F-12 were added into each well of 96-well plates. CCK8 cell proliferation assays were performed by measuring absorbance at 450 nm by using a microplate reader (Thermo Fisher, USA) after incubation at 37 °C for 1–4 h. Detailed grouping information is shown in Table S2. The specified inhibitors above include deferoxamine mesylate, Ferrostatin-1 (MedChemExpress, #HY-100579), Necrostatin-1 (#HY-15760), Z-VAD-FMK(#HY-16658B), and 3-methyladenine(#HY-19312).

### Chemiluminescence

ROS assay kit (#E004-1-1) of Nanjing Jiancheng Bioengineering Institute was used to detect the intracellular ROS level. The lipid peroxidation level was detected using a C11 BODIPY 581/591 fluorescent probe (#MX5211) from Shanghai Maokang Biotechnology Co., Ltd. Far-red Labile Fe^2+^ live cell dye (BioTracker, #SCT037) was used to detect Fe^2+^ level. All operations were performed according to the manufacturer’s instructions.

### Isolation of exosomes

Mouse granulosa cells were acclimated in DMEM/F12 without exosomes for 24 h, and then subjected to different interventions. No special treatment was provided to the control group (CON). The ferric citrate (FAC) group was treated with 100 μM ferric citrate. The deferoxamine mesylate (DFO) group was treated with ferric citrate (100 μM) and deferoxamine mesylate (100 μM). VE group was treated with ferric citrate (100 μM) and VITE (200 μM). All groups were cultured for 48 h. After the termination of incubation, centrifugation was carried out in steps at 4 °C and 300 × *g* for 10 min to remove cells and 3000 × *g* for 10 min to remove large cell debris. After centrifugation at 10,000 × *g* for 40 min, the supernatant was filtered through a 0.22 μM PVDF filter (Millipore, USA). Finally, centrifugation at 100,000 × *g* for 90 min, the supernatant was removed and resuspended in 200 μL of PBS to obtain the cell supernatant exosomes precipitation.

### Nanoparticle tracking analysis

Exosomes were diluted in PBS and analyzed using nanoparticle tracking analyzer (Particle Metrix, German, ZetaVIEW®). The instrument was equipped with a 405 nm excitation laser and pre-calibrated with a 100 nm PSL reference standard for the concentration of exosomes. All nanoparticle tracking analysis measurements used exactly the same camera settings and identical tracking parameters. The recommended parameters for extracellular vesicle detection are sensitivity of 85, shutter of 70, minimum brightness of 20, minimum size of 10, and maximum size of 200. Videos were captured at 30 frames per second and analyzed for size and concentration by using ZetaView software (Particle Metrix).

### RNA-sequencing analysis

The culture medium containing 20% EMFF or 20% COFF was added to KGN. Total RNA was isolated using TRIzol Reagent (Invitrogen, Breda, The Netherlands). mRNA was extracted from six replicate samples per group. The constructed mRNA libraries were QC qualified on the 2100 Bioanalyzer (Agilent, Beijing, China) and sequenced using Illumina HiSeq2500 (NEB, USA). Kyoto Encyclopedia of Genes and Genomes (KEGG) pathway analysis was conducted using the KEGG pathway database to explore the potential gene involved in ferroptosis and ferritinophagy. GSEA analysis was conducted using the GSEA database to further explore ferroptosis-related gene expression differences between EMFF and COFF group.

RNA sequencing of small RNA of exosomes from granulosa cells was done and analyzed. miRNAs of exosomes were extracted using the exoEasy Maxi kit (QIAGEN) and quantified using Qubit 2.0 (Life Technologies). The integrity was confirmed using Agilent 2100 TapeStation (Agilent). Small RNA sequencing was conducted using Illumina HiSeq 2500 with read lengths of 15–41 bp.

### Transmission electron microscopy

The cells to be observed were fixed with 2.5% normal glutaraldehyde in PBS, aspirated into the centrifuge tube, and centrifugated at 2000 rpm for 2 min. The fixative was discarded followed by a new fixative for electron microscopy, and the cells were observed under TEM (Hitachi).

Exosome samples were transferred into 200-mesh Formvar and carbon-coated copper grid (Ted Pella) and incubated for 1 min. After adsorption, the grid was rinsed with water, and the excess solution was removed. Grids were then stained with 1% phosphotungstic acid for 1 min, and the excess solution was cleared. Exosomes were observed by TEM (FEI Tecnai G2 Spirit BioTWIN) after drying at room temperature.

### Detection of follicular fluid-related indicators

Iron detection kit (#A039-1-1) was obtained from Nanjing Jiancheng Bioengineering Institute. The iron content in FF was detected using colorimetric method. The treated supernatant was collected in a microplate reader to detect the OD value of absorbance in each tube with an optical diameter of 0.5 cm and wavelength of 520 nm. Transferrin assay kit (#E-80TX) was obtained from Immunology Consultants Laboratory, Inc. The content of TF in FF was determined by the immunoperoxidase method. Hepcidin detection ELISA kit (#E4692-100) was obtained from Biovision (California, USA). All the above operations were performed according to the manufacturer’s instructions.

### Detection of ferroptosis-related indicators

GPX4 assay kit (CSB-EL009869HU) was obtained from CUSABIO (Hubei, China). Total GSH/oxidized glutathione assay kit (#A061-1) and MDA assay kit (#A003-4-1) were obtained from Nanjing Jiancheng Bioengineering Institute. The above operations were performed according to the manufacturer’s instructions.

### Real-time qPCR

The mRNA transcript levels of TF, ferroportin1 (FPN1), FTL, FTH1, NCOA4, HERC2, ATG 5, and ATG7 in human granulosa cells were analyzed by RT-PCR. Primer sequences are described in Table S3. Total RNA was extracted using TRIzol reagent and quantified spectrophotometrically at 260 nm. Real-time PCR was performed using LightCycler® 480II (Roche, Basel, Switzerland). The qPCR protocol consisted of initial denaturation at 95 °C for 30 s, 40 cycles of amplification at 95 °C for 5 s, 60 °C for 30 s, and a final melting curve stage. mRNA levels were calculated using the 2-ΔΔCT method and normalized to glyceraldehyde-3-phosphate dehydrogenase (GAPDH) levels.

### Western blot analysis

Western blot analysis was performed according to the standard procedure to analyze the expression of FTH1 (1:1000, BOSTER, California, USA, #BM4487), NCOA4 (1:1000, Abcam, Cambridge, MA, USA, #ab86707), ATG5 (1:1000, BOSTER, #BA3525-2), β-actin (1:2000, Abcam, #ab8226), TP53 (1:1000, BOSTER, #BM4309), CD63 (1:1000, Abcam, #ab193349), CD9 (1:1000, Abcam, #ab92726), and GAPDH (1:1000, Shanghai Siding Biotechnology Co., Ltd., China, #SD0033).

### NCOA4-overexpressing and -silent

ADV6 and homo NCOA4 plasmids were obtained from Shanghai GenePharma Co., Ltd. (Shanghai, China). NCOA4-overexpressing KGNs were obtained by transfection of ADV6-Homo NCOA4 plasmid, and the results were confirmed by RT-PCR analysis. The RNA oligo (dT) of NCOA4 and GP-transfect-Mate transfection reagents were provided by Shanghai GenePharma Co., Ltd. The RNA oligo (dT) sequences are shown in Table S4. After configuration of the solution as per instructions, the solution was added dropwise to six-well plates containing KGN. NCOA4-silent KGN was finally obtained by examining mRNA after 24–72 h of transfection and protein levels after 48–96 h at 37 °C.

After 48 h of intervention with physiological concentrations of ferric citrate, NCOA4-overexpressing and -silent KGN were subjected to lipid peroxidation staining with C11 BODIPY 581/591 fluorescent probe, as described previously. DAPI staining solution (Beyotime, Shanghai, China, #C1005) was subsequently added to cover the cells for 3–5 min at room temperature. Finally, we removed DAPI and rinsed it thrice with PBS, and KGN was observed under a fluorescence microscope (OLYMPUS, Japan).

### Morphological observations of oocytes

The ovarian tissue was minced well and filtered through 100- and 40-μM mesh (BD Falcon), and cells in the upper 40-μM mesh were collected. Oocytes were obtained by resuspension in pre-warmed KSOM mouse embryo culture medium after centrifugation at 1000 rpm for 5 min. Subsequently, oocytes were seeded in 96-well plates and placed in an incubator at 37 °C with 5% CO_2_ and were intervened by exosomes from different groups above for 6 h. Oocyte morphology was observed microscopically and recorded. Investigators were blinded to the group allocation when assessing the results.

### Serum measurements

Mouse serum was detected using mouse E_2_ ELISA Kit (#E-90-96), FSH ELISA Kit (#E-86-96), and LH ELISA Kit (# E-85-96) from Genfine Biological Technology Co., Ltd (Beijing, China). The specific experimental procedures were performed according to the manufacturer’s instructions.

### Histological assessments

Ovarian tissue samples were fixed in 10% neutral formalin solution overnight, embedded in paraffin, and cut into 4-μM thick sections. Subsequently, the sections were stained with hematoxylin and eosin (H&E) according to standard protocols. Light microscopy was performed to observe the results and acquire images.

### Prussian blue staining and immunohistochemistry

All mouse ovarian specimens were fixed with 4% formaldehyde, embedded in paraffin, and cut into 5-μM thick sections. Following paraffin removal and dehydration, sections were incubated in 3% hydrogen peroxide for 30 min to quench endogenous non-specific peroxidase activity. For Prussian blue staining, slides were immersed for 15–25 min in a mixed solution of equal parts of 2% potassium ferrocyanide and 2% hydrochloric acid. For immunohistochemistry, operations were conducted following standard protocols. Samples were incubated with anti-GPX4 antibody (1:400, BOSTER, #BA3802-1) overnight at 4 °C and subsequently rinsed with PBS. Samples were then incubated with HRP-labeled anti-rabbit/mouse secondary antibody (Shanghai WellBio technology Co., Ltd., China, #WB0177 / #WB0176) for 45 min at 37 °C. Finally, all slides were incubated with 3,3-diaminobenzidine tetrahydrochloride (Maxim, China) for 6 min and counterstained with hematoxylin. After absolute ethanol dehydration and neutral gum sealing, the specimens were observed and photographed under a microscope (Leica, German).

### Statistical analyses

Data were analyzed using GraphPad Prism software (v.8.1.2, La Jolla, CA, USA). Analysis was performed using Student’s *t* test between two groups and one-way ANOVA among three or multiple groups. We statistically compared the similar variances between the groups as well. All analyses were conducted with SPSS 21.0 software. Statistical significance was considered at *P* < 0.05.

## Supplementary information

Supplementary Materials

## Data Availability

All sequencing profiles, including RNA-seq and miRNA-seq, were uploaded to GEO (GSE205494, GSE205579).
