# Supplementary material for: Iron-overloaded follicular fluid increases the risk of endometriosis-related infertility by triggering granulosa cell ferroptosis and oocyte dysmaturity
Source: Cell Death Dis. 2022 Jul 4;13(7):579. doi: 10.1038/s41419-022-05037-8 (PMC9253011; doi:10.1038/s41419-022-05037-8)
Supplement: Supplementary file 1 — Supplementary Materials [file 41419_2022_5037_MOESM1_ESM.docx]

**Supplementary Material**

**Inclusion and exclusion criteria**

The inclusion criteria for patients with EMs-related infertility were pathological diagnosis of EMs with the absence of natural conception for more than 1 year without contraception and with normal routine semen analysis (according to the Kruger criteria) of the spouse male. The key inclusion criteria for control group were women undergoing assisted reproduction caused by spouse male infertility. The male infertility diagnosis standard was based on the *World Health Organization standardized examination and diagnosis manual for male infertility*.

The key exclusion criteria were the presence of disorders of ovulation such as polycystic ovary syndrome, adenomyosis, hyperthyroidism, systemic lupus erythematosus, or other autoimmune diseases, immunodeficiency virus infection or any active infection, infertility of tubal factors, use of hormonal or anti-inflammatory medications within 3 months prior to presentation, contraindications to any ovarian stimulation treatment, smoking, and absence of historical data.

**Supplementary Tables**

**Supplementary Table 1. Diet composition in three groups**

| **Composition and proportion** | **Standard iron diet** | **Low iron diet** | **High iron diet** |
| --- | --- | --- | --- |
| Casein | 20.0% | 20.0% | 20.0% |
| Corn starch | 15.0% | 15.0% | 15.0% |
| Sucrose | 50.0% | 50.0% | 49.2% |
| Cellulose | 5.0% | 5.0% | 5.0% |
| Corn oil | 5.0% | 5.0% | 5.0% |
| Normal iron ore material | 3.5% | / | 3.5% |
| Iron-deficient minerals | / | 3.5% | / |
| Ferrous sulfate | / | / | 0.79% |

**Supplementary Table 2. Cell viability measurement grouping**

| **Group** | **Intervention** |
| --- | --- |
| CON | DMEM / F12 medium |
| COFF | 20% control FF |
| EMFF | 20% FF in EMs-related infertility (EMFF) |
| EMFF+DFO | 20% EMFF + Deferoxamine (100 μM) |
| EMFF+FER | 20% EMFF + Ferrostatin-1 (60 nM) |
| EMFF+NEC | 20% EMFF + Necrostatin-1 (0.5 μM) |
| EMFF+ZDF  EMFF+ME | 20% EMFF + Z-DEVD-FMK (20 μM)  20% EMFF + 3-Methyladenine (0.5 μM) |

**Supplementary Table 3. Primers of real-time qPCR**

| **Primer** |  | **Primer sequences（5'-3')** |
| --- | --- | --- |
| TF-human | Forward Primer | GGTGGCAGAGTTCTATGGGTC |
|  | Reverse Primer | ACAGTAAAGTAAGCCTATGGGGA |
| FPN1-human | Forward Primer | TGGATGGGTTCTCACTTCCTG |
|  | Reverse Primer | GTCAATCCTTCGTATTGTGGCAT |
| FTH1-human | Forward Primer | CAGAACTACCACCAGGACTCA |
|  | Reverse Primer | CACATCATCGCGGTCAAAGT |
| FTL-human | Forward Primer | CCAGCACCGTTTTTGTGGTT |
|  | Reverse Primer | CAATTCGCGGAAGAAGTGGC |
| ATG5-human | Forward Primer | AAGCAACTCTGGATGGGATTG |
|  | Reverse Primer | TCCTAGTGTGTGCAACTGTCC |
| ATG7-human | Forward Primer | CCAGAAAGGAGGCATGGGAC |
|  | Reverse Primer | GCCTCACAGGATTGGAGTAGG |
| NCOA4-human | Forward Primer | ACAGTTGCATAAGCCGTCACC |
|  | Reverse Primer | TGAGCCTGCTGTTGAAGTGTC |
| HERC2-human | Forward Primer | GCGCTGTCTTTTGCCTTTG |
|  | Reverse Primer | AGGAACCTGGTCGCTCTCTC |
| GAPDH-human | Forward Primer | AAAATCAAGTGGGGCGATGC |
|  | Reverse Primer | TGGTTCACACCCATGACGAA |

**Supplementary Table 4. RNA oligo (dT) sequence**

| **Gene** | **Sequence** | |
| --- | --- | --- |
|  | **Sense (5'**–**3')** | **Antisense (5'**–**3')** |
| NCOA4-homo-105 | GGGCUGAACAGCAAAUUAATT | UUAAUUUGCUGUUCAGCCCTT |
| NCOA4-homo-1789 | GCAGCUUAAAGUUGAUAAATT | UUUAUCAACUUUAAGCUGCTT |
| NCOA4-homo-1551 | CUGGCAAACAGAAGUUUAATT | UUAAACUUCUGUUUGCCAGTT |
| Negative control | UUCUCCGAACGUGUCACGUTT | ACGUGACACGUUCGGAGAATT |

**Supplementary Figures**


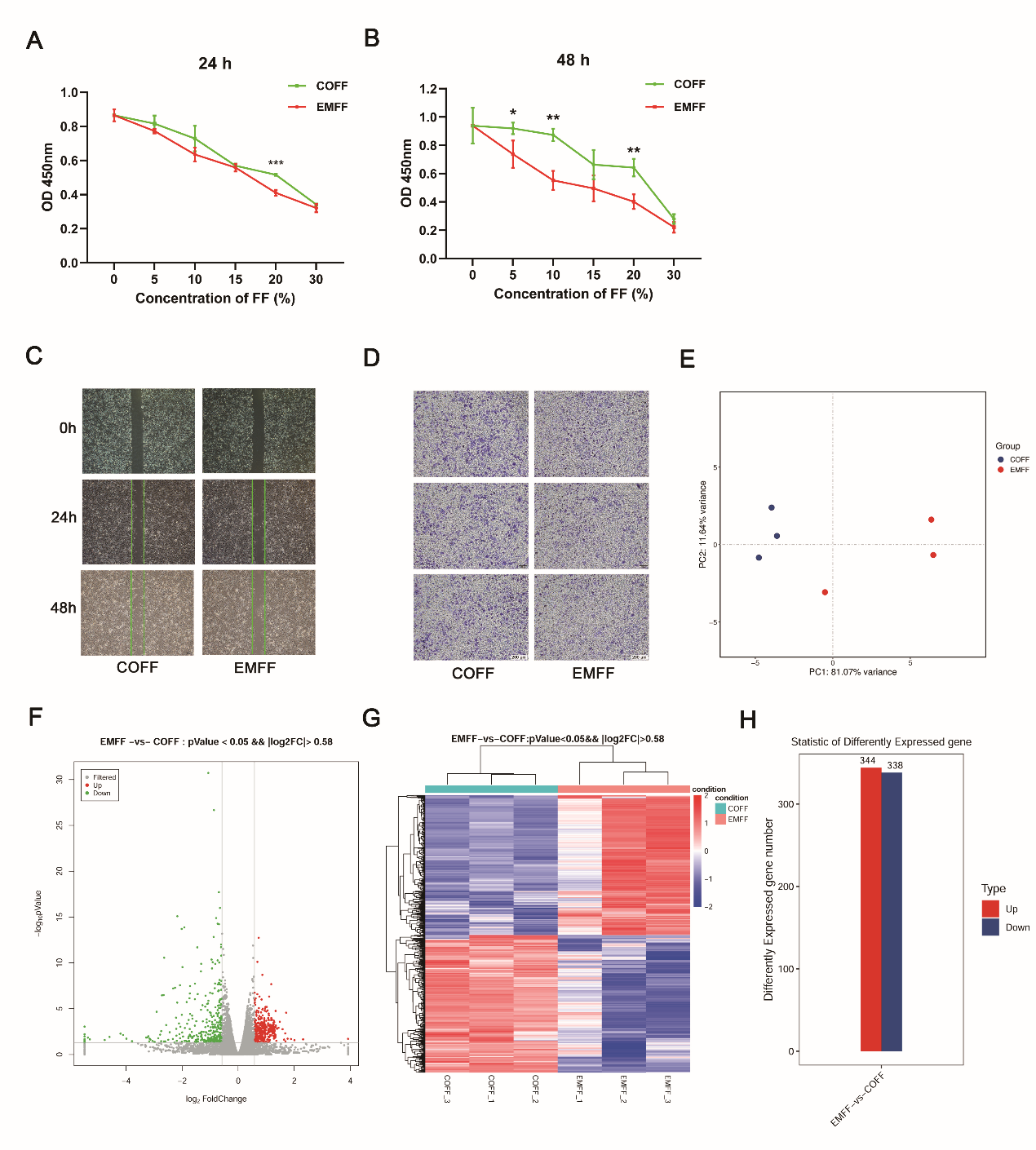


**Supplementary Figure 1**

**KGN treated with EMFF and COFF**. (A-B) KGN cell viability with 0%, 5%, 10%, 15%, 20%, and 30% EMFF and COFF treatment at 24 and 48 h. Data are expressed as means ± SD and analyzed by Student’s *t* test. **P* < 0.05, ***P* < 0.01, and ****P* < 0.001. (C-D) Representative pictures of scratch assay graphs and migration experiment graphs of KGN cells after COFF and EMFF intervention. Scale bar = 200 µm. (E) Principal component analysis plot between EMFF (*n* = 3) and COFF (*n* = 3). (F) Volcano plot of differentially expressed genes in the EMFF and COFF group (differential fold > 1.5, *P* <0.05). The horizontal axis is log_2_ (fold change), and the vertical axis is the – log_10_p-value. (G) Heatmap of differential genes in KGN treated with COFF (*n* = 3) and EMFF (*n* = 3). (H) Statistical histogram of differentially expressed genes between the COFF and EMFF group.


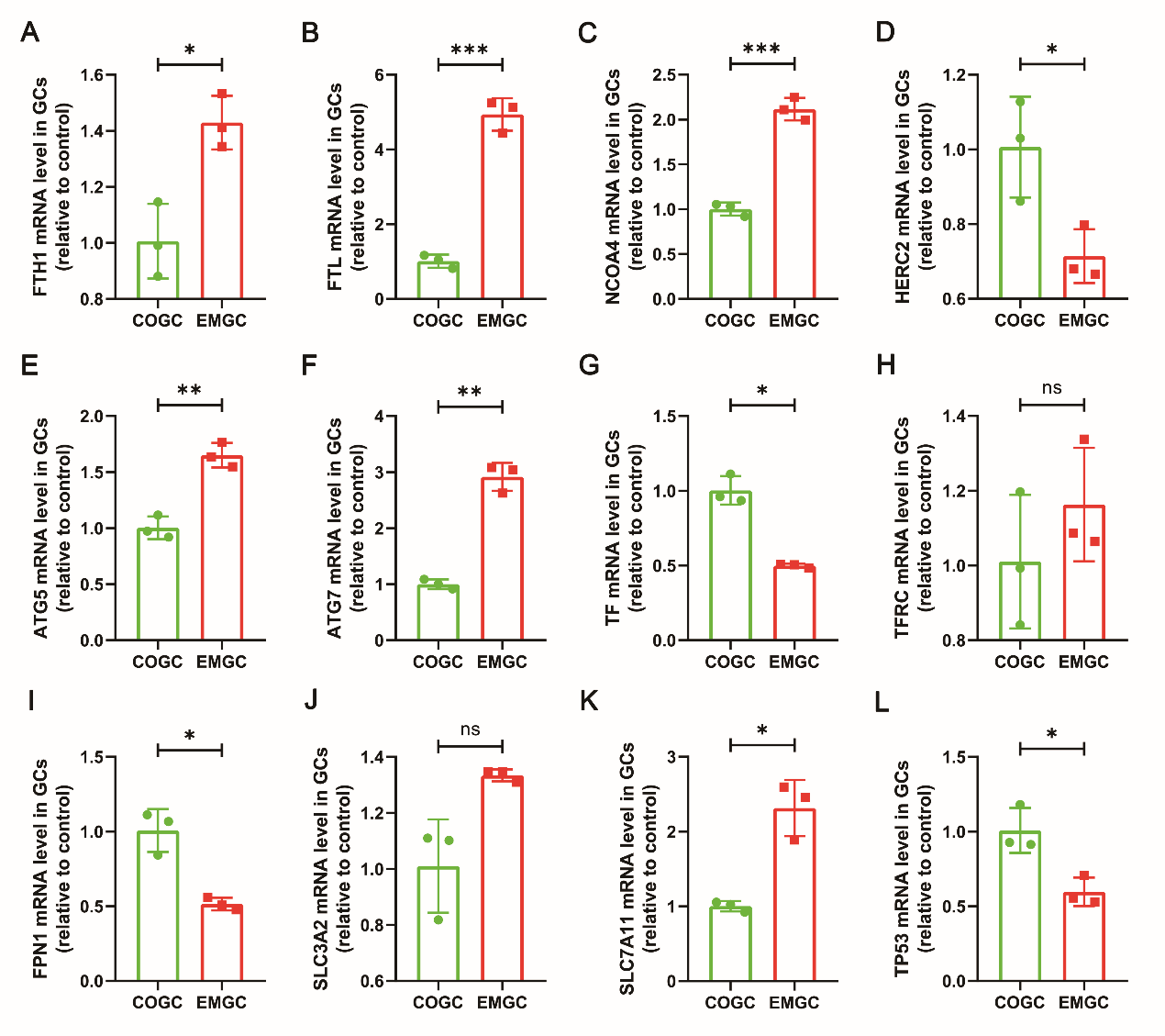


**Supplementary Figure 2**

**The mRNA levels of ferroptosis and ferritinophagy-related proteins in granulosa cell from EMs-related infertile patients and the control group.** Data are expressed as means ± SD and analyzed by Student’s *t* test (*n* = 3). **P* < 0.05, ***P* < 0.01, ****P* < 0.001, and ns, no significance.


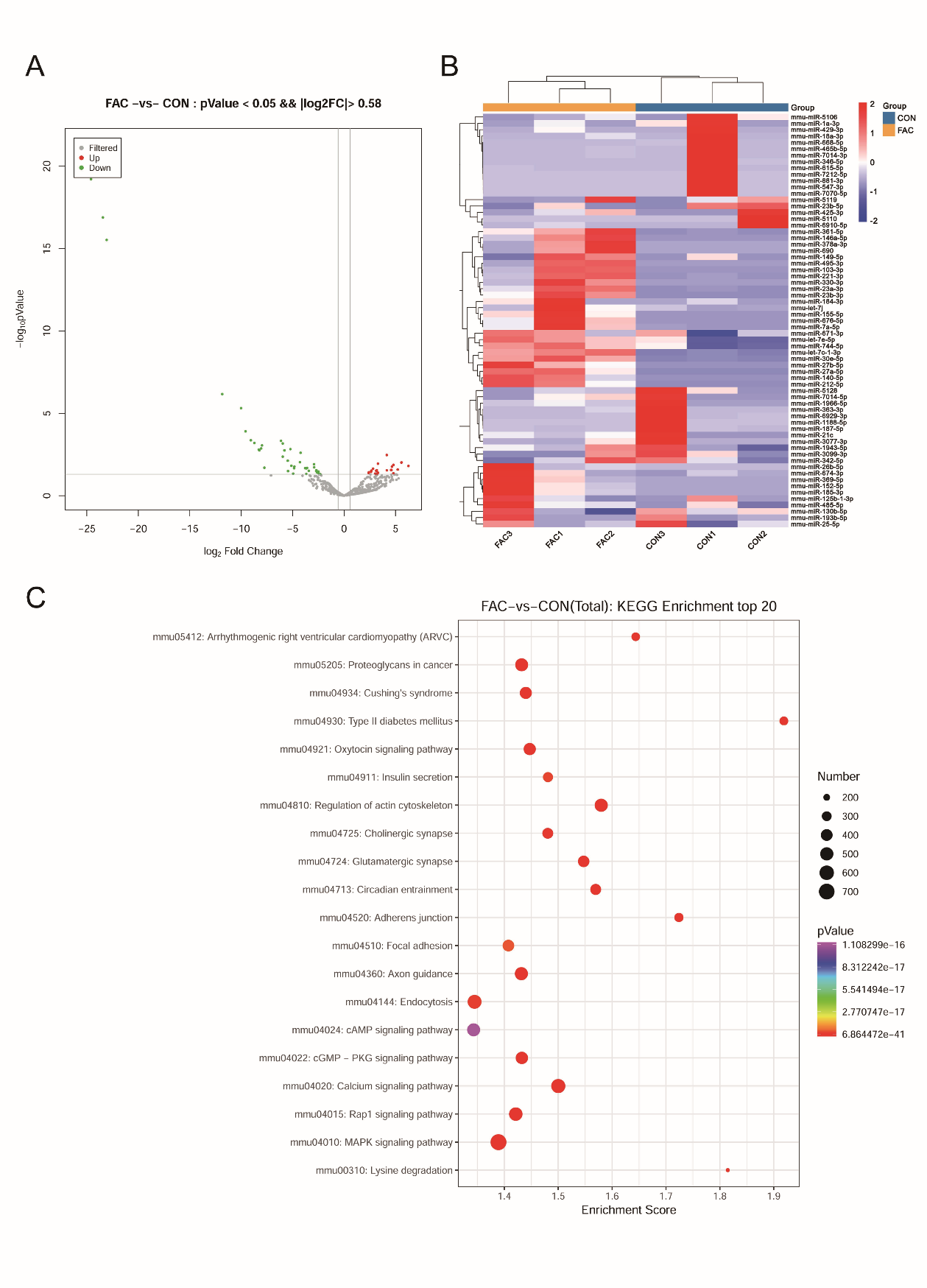


**Supplementary Figure 3**

**Differential miRNAs in exosomes of granulosa cell from ferric citrate intervention and control group.** (A) Difference volcano plot, in which grey indicates non-differential miRNAs, red indicates upregulated significant differential miRNAs, and green indicates downregulated significant differential miRNAs (differential fold > 1.5, *P* <0.05); X-axis shows the log_2_fold change, and the Y-axis shows the –log_10_p-value. (B) Heatmap of differential miRNA expression between the CON group (*n* = 3) and the FAC group (*n* = 3). (C) Top 20 bubble plot of KEGG enrichment analysis of differential miRNA target genes between the CON and FAC group. Sort by –log_10_Pvalue corresponding to each entry. Each dot in the figure corresponds to a pathway; the smaller the p-value, the more color tends to red; larger dots indicate more genes in the pathway.
